# Transcriptomic analysis of cork during seasonal growth highlights regulatory and developmental processes from phellogen to phellem formation

**DOI:** 10.1038/s41598-021-90938-5

**Published:** 2021-06-08

**Authors:** Sandra Fernández-Piñán, Pau Boher, Marçal Soler, Mercè Figueras, Olga Serra

**Affiliations:** Laboratori del Suro, Departament de Biologia, Universitat de Girona, Campus Montilivi, 17003 Girona, Spain

**Keywords:** Plant development, Cell fate, Plant stem cell

## Abstract

The phellogen or cork cambium stem cells that divide periclinally and outwardly specify phellem or cork. Despite the vital importance of phellem in protecting the radially-growing plant organs and wounded tissues, practically only the suberin biosynthetic process has been studied molecularly so far. Since cork oak (*Quercus suber*) phellogen is seasonally activated and its proliferation and specification to phellem cells is a continuous developmental process, the differentially expressed genes during the cork seasonal growth served us to identify molecular processes embracing from phellogen to mature differentiated phellem cell. At the beginning of cork growth (April), cell cycle regulation, meristem proliferation and maintenance and processes triggering cell differentiation were upregulated, showing an enrichment of phellogenic cells from which phellem cells are specified. Instead, at maximum (June) and advanced (July) cork growth, metabolic processes paralleling the phellem cell chemical composition, such as the biosynthesis of suberin, lignin, triterpenes and soluble aromatic compounds, were upregulated. Particularly in July, polysaccharides- and lignin-related secondary cell wall processes presented a maximal expression, indicating a cell wall reinforcement in the later stages of cork formation, presumably related with the initiation of latecork development. The putative function of relevant genes identified are discussed in the context of phellem ontogeny.

## Introduction

Radial growth (secondary growth) of plant organs, either herbaceous or woody plants, angiosperms or gymnosperms, involves the activation of two secondary meristems: the cambium (or vascular cambium) and phellogen (or cork cambium). Both meristems constitute a bifacial stem cell population from which derivatives are formed by periclinal divisions and specified on opposing sides. Secondary growth is initiated by vascular cambium, which generates vascular tissue (xylem inwardly and phloem outwardly) and results in stem and root thickening. Due to the vascular radial growth, the primary protective tissues (epidermis and endodermis) break and a phellogen formed inwardly develops a complete periderm conferring protection to the mature organs^1^. Periderm is also generated in the exposed surfaces of the abscission zones of plants, and in response to mechanical injury or parasite attack forming a wound periderm^2^.

The periderm ontogeny is initiated by the periclinal division of the phellogen stem cells, which provide the derivatives at opposing sides that will specify to phellem (outwardly) and phelloderm (inwardly). The three layers, phellogen, phellem (or cork) and phelloderm constitute the periderm^2^. The newly formed phellem cell elongates outwardly in the radial direction while its cell wall thickens as a consequence of accumulating structural components. In addition to the polysaccharide primary cell wall, the phellem cell wall deposits suberin and lignin polymers, and also some soluble secondary metabolites such as extractives (fatty-acyl derived and triterpenes) and tannins^3,4^. This specialized cell wall provides a waterproofing layer that in turn isolate the cell itself from water and nutrients, thus dying at maturity. These phellem cells are usually forming a multilayered tissue which provide altogether the protective function^2^.

**Figure 1:**
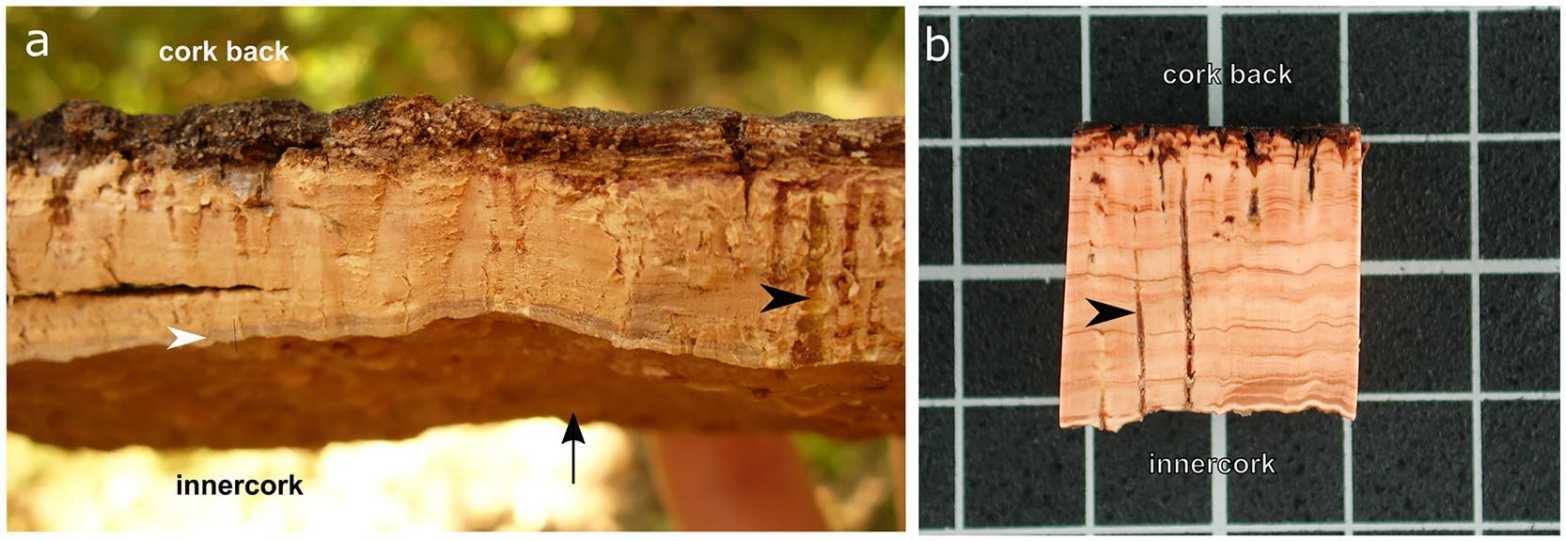
Anatomy of cork plank (outer bark) from *Quercus suber.* (**a**) Cork freshly-harvested at the late spring (June) in Romanyà de la Selva (Girona, Spain) showing this year produced living tissue (white arrowhead) in the innercork side, which faces the phellogen (black arrow). (**b**) A dried and polished sample is shown to better appreciate the annual cork-rings seen as lighter (earlycork) and darker brown (latecork) zones. Lenticels are highlighted with a black arrowhead. The grid scale distance in B corresponds to 1 cm. The figure was constructed using the free and open source Inkscape software 1.0 (https://inkscape.org/).

In most woody plants, the-each-year periderm is replaced by a new periderm formed underneath the previous one. However, a few exceptions exist, notably cork oak (*Quercus suber*), which naturally produces a unique and single periderm throughout the entire tree life. The cork oak permanent and continuous phellogen cylinder presents seasonal growth, which through activation-inactivation series adheres the new formed phellem cells to the death phellem cells of the previous years^5^, hence providing a pure outer bark of thick phellem/cork. The fact that the phellogen layer forms a continuous cylinder lengthwise the tree trunk allows the cork harvesting as an entire plank by applying a moderate tensile force. This is possible when phellogen is fully active and has produced a set of phellem cells with also thin and fragile cell walls (usually June)^6,7^. In the plank, the phellogen and new phellem cells are in the innercork side and the old and death phellem cells of previous years are extended outwardly to the cork back side (Fig. 1). The first cork produced by cork oak, known as virgin cork, is irregular in structure, density and thickness and is hard-rough. The following harvested corks, extracted sequentially every 9–12 years, the time required to allow the tree to re-grow its outer bark, are known as reproduction cork. Although all the cork planks are exploited commercially as raw material for *i.e.* agglomerates, only the third (2nd reproduction cork) and successive harvests can meet the regular structure with smooth and unblemished bark to present the quality required for stopper production. This best quality reproduction cork is termed amadia cork^8^.

The seasonal growth of cork, starting around April, produces two cork cell populations. From April to July cork expands at a high rate producing the earlycork^9^, being around June the highest growth rate. At the cellular level, it is when the phellogen and phellem are fully active: the rate of cell division and cell expansion is high and the resulting phellem cells (earlycork cells) will be numerous (40 to 200 cells per annual row), long and with thin walls. Advancing to the end of the growing season, the cell division and expansion rates decrease, resulting in the formation of few cork cells (4–8 cells per annual row) with thicker walls (latecork cells)^4,10^. These phellem cellular differences result in the observation of annual-cork rings (Fig. 1), which includes the earlycork and latecork subsequently accumulated in successive years^11^.

Most of the processes essential for phellem formation and development are mainly unknown. However, a few genes involved in suberin biosynthesis, transport and regulation, mostly identified as preferentially expressed in cork oak or poplar phellem^5,12^, have been functionally characterized in potato tuber phellem (skin) or in Arabidopsis root endodermis and seeds^13^. This has evidenced that most suberized tissues, including phellem, use the same protein network. In the same line, phellogen and vascular cambium, both bifacial secondary meristems with seasonal growth, commonly upregulates a set of specific genes not present in other tree trunk tissues^14^, pointing that both meristems share molecular mechanisms for its formation and maintenance. The shared mechanisms have been evidenced very recently, since the vascular cambium master regulators WUSCHEL-RELATED HOMEOBOX 4 (WOX4) and KNAT1/BREVIPEDICELLUS (BP) were also found to control the Arabidopsis root phellogen activity^15^.

In this work, we wanted to understand the molecular mechanisms expanding from the phellogen derivative formation to the phellem cell specification and differentiation. To that, we analysed the transcriptome of the newly formed cork cells (living phellem cells or phelloid) isolated from innercork planks at three different time points of the growing season: at the beginning of the cork growing season (April), at the maximum cork growth rate (June)^6,7^ and at advanced stage (July). The transcriptome seasonal variation was used as a readout of the molecular processes involved in phellem cell ontogeny. The main processes and candidate genes highlighted are involved in the phellogen stem cell identity and phellem differentiation, while others may be key players in the phellem cell wall modification.

## Results

### High-throughput gene expression profiling of innercork along the growing season

To understand the changes produced in cork transcriptome during the growing season, cork samples were collected in April, June and July from three to four different trees (biological replicates). Different trees were used for April, June and July sample collection, amounting to a total of 11 trees. The eleven corresponding RNA samples were sequenced by Illumina HiSeq2500 sequencing technology. The tissue used for RNA extraction was the newly-produced and living cork tissue from the innerbark of the plank, adjacent to the phellogen tissue (Fig. 1). A total of 568 × 10^6^ reads with an average length of 122 bp were obtained. On average, 81% of the reads uniquely mapped against the cork oak genome (GCF_002906115.1 (CorkOak1.0))^16^, identifying 32,024 *Q. suber* active loci (Supplemental Table 1). For each gene, the normalized read count was calculated obtaining for the whole transcriptome the gene expression profile along the cork growing season (Supplemental Data Set 1). For functional characterization, 30,676 *Q. suber* genes were annotated to the corresponding Arabidopsis most similar gene, which has been considered the homolog throughout the manuscript (TAIR10 protein database; BLASTP E-value < 10^–5^) (Supplemental Table 1 and Supplemental Data Set 1).

To validate the expression values of RNA-seq data, the log_2_Fold-Change (FC) values obtained in this RNA-seq experiment for 29 genes representative of processes described for cork development were compared with the log_2_FC values obtained from new and previously published RT-qPCR analyses which used the same RNA samples (Supplemental Table 2)^7,17^. A significant Pearson correlation (p < 0.001) in all pair-wise comparisons (June/April, July/April and July/June) was observed (Supplemental Figure 1) validating the RNA-seq results.

### The innercork samples contained transcripts from phellem and phellogen

When the cork plank is extracted, it breaks along the phellogenic tissue, therefore the RNA extracted was hypothesized to contain transcripts from living phellem cells but also phellogen. To understand at which extent phellem and phellogen were represented in the tissue collected, we seek in the cork transcriptome for those genes described to be specifically and differentially expressed in phellem and phellogen/phelloderm of *Betula pendula* (birch) tree trunk^14^, using the corresponding Arabidopsis gene with higher similarity. The cork transcriptome expressed the 88.17% of genes identified as differentially expressed in birch phellem, but also the 79.90% of the phellogen/phelloderm (padj < 0.01 and a fold change -1 > log_2_FC > 1) (Supplemental Table 3). These results suggest that the cork samples we used also contained phellogen cells.

### Most genes involved in suberin deposition are induced in June

**Figure 2:**
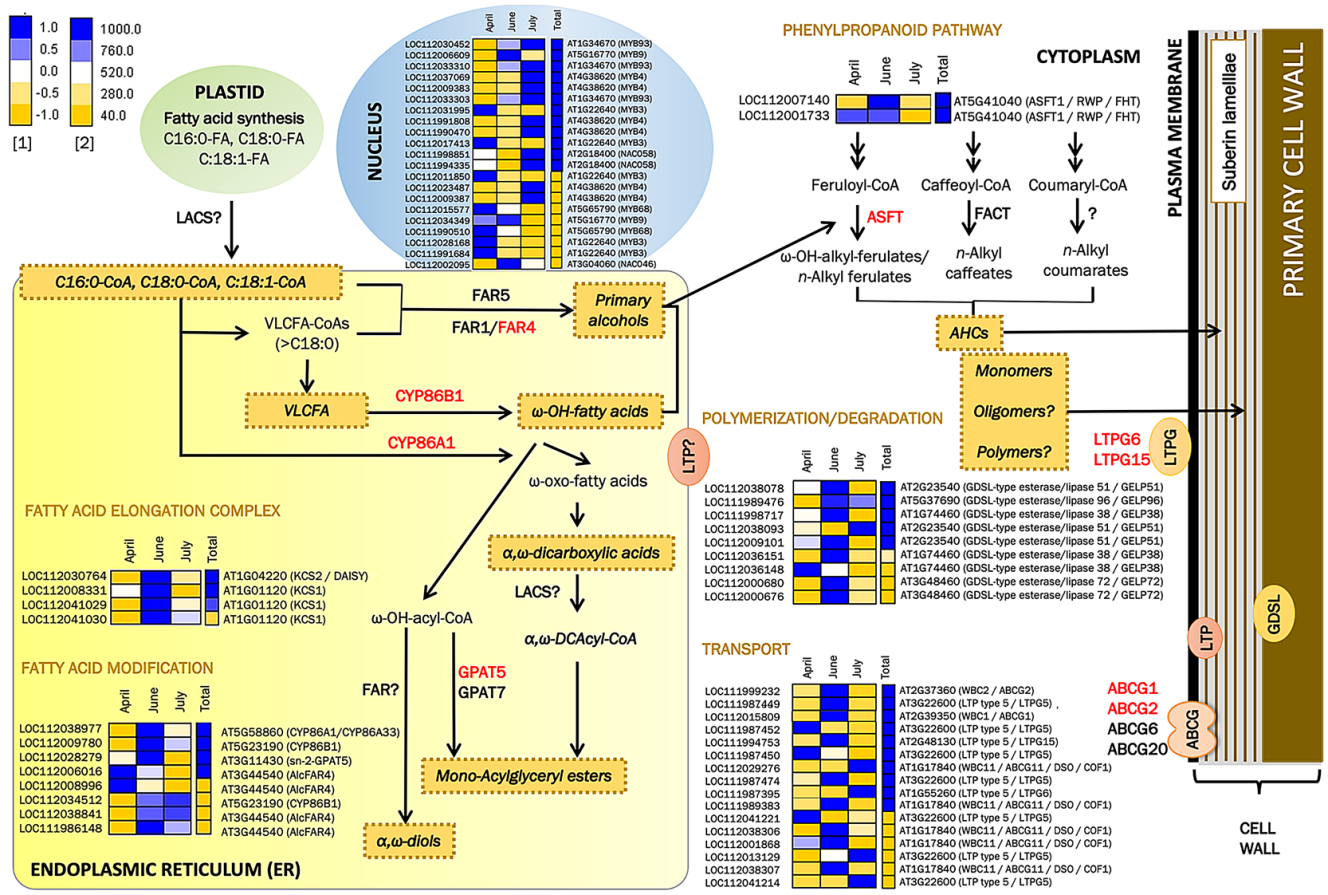
Expression pattern along the cork growing season of genes involved in suberin-related processes. For each gene, the relative expression pattern (April, June and July)^1^ and the total expression^2^ of normalized read counts are shown as a heatmap, in which blue and yellow colours represent high and low expression values, respectively. The genes with demonstrated function identified in the cork transcriptome are highlighted in red. AHCs = alkyl hydroxycinnamates; VLCFA = very long chain fatty acid. The heatmaps were constructed using the EXPANDERS software^75^.

Since suberin is a key structural component deposited in the phellem cell wall during its development, we followed a targeted approach in which the expression pattern of suberin related genes was evaluated along the growing season. First, we identified in the cork transcriptome 68 *Q. suber* genes that corresponded to 25 Arabidopsis homologs involved in suberin and/or suberin-associated wax accumulation classified on AraLip database (http://aralip.plantbiology.msu.edu/pathways/pathways) or reported more recently in literature (Supplemental Table 4A). These data suggest putative duplications of these genes in *Q. suber* genome. The identified suberin-related genes, based on the enzymatic or regulatory function of the encoded protein, were depicted and classified within cell compartments and metabolic pathways/processes active in a suberizing cell, namely fatty acid elongation complex (FAE), fatty acid modification, transport to the cell wall, phenylpropanoid pathway and transcription factors (Supplemental Table 4A and Fig. 2). For each *Q. suber* transcript, a heatmap shows the relative expression along the cork growing season (April, June and July) and the total expression.

Importantly, 58.33% (21 of 36) of the genes with highest expression (> 760 normalized read counts) in cork, including the 15 of the top 16, showed a maximum of expression in June, and included genes involved in suberin biosynthesis (CYP86A1, CYP86B1, GPAT5, KCS2, KCS1 and ASFT), polymerization (GELP38, GELP51 and GELP96), transport to the cell wall (ABCG1, ABCG2, ABCG11, LTPG5 and LTPG15) and positive regulation (MYB9) (see Supplemental Table 4A for references). These results point that, although the phellem cells are committed to suberin production along the entire growing season, it is in June when there is a more intense suberization since cork tissue is enriched in phellem cells actively suberizing.

To seek for new candidate suberin genes, the complete AraLip database was compared with the cork transcriptome. We identified 207 *Q. suber* genes (110 Arabidopsis homologs) not previously related with suberin (Supplemental Table 4B), which were classified into the previously mentioned suberin functional categories and the fatty acid precursor synthesis in the plastids (Supplemental Figure 2). Based on the expression profile of suberin-known genes, the best candidates were those showing highest expression in cork tissue with a maximum in June.

### Differential expression analyses highlight seasonal transcriptional patterns during cork formation

**Figure 3:**
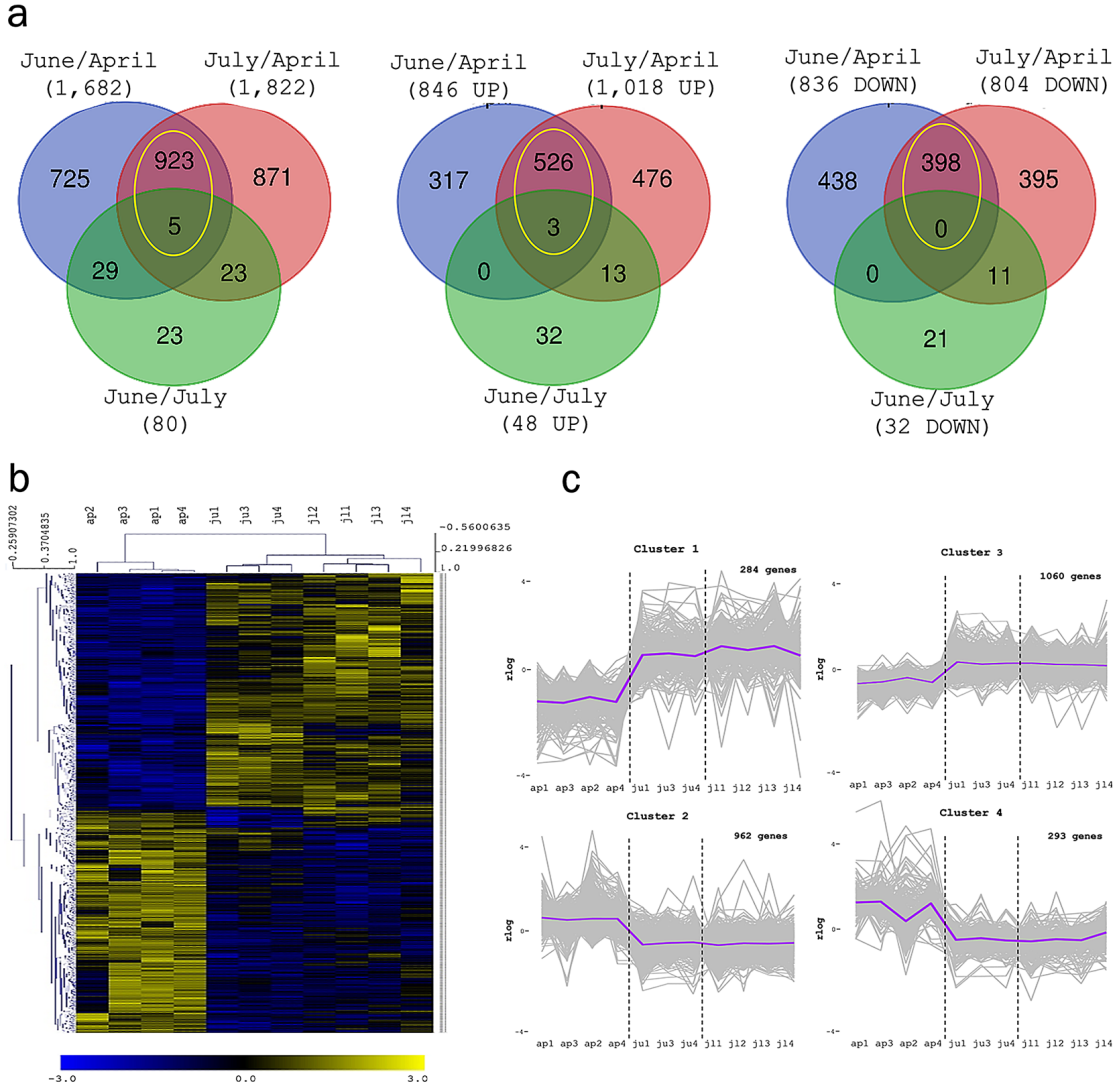
Differential expression along the cork growing season. (**a**) Venn diagrams showing DEGs, upregulated (UP) and downregulated (DOWN), between June/April, July/April and June/July. The yellow circle includes the genes identified in both June/April and July/April comparisons. (**b**) Hierarchical clustering of cork samples based on the expression of DEG along the growing season. C. K-means clusters of co-expressed genes in April, June and July. Cluster 1 and 3 contain genes downregulated in April and upregulated in June and July, whereas cluster 2 and 4 showed the opposite pattern containing genes upregulated in April and downregulated in June and July.

To identify the genes regulated during the growing season, the gene expression at the different cork growing stages were pair-wised (June/April, July/April and July/June) compared using the DESeq2 statistical package. DEGs were selected using a filter of padj < 0.01 and absolute log_2_Fold-Change > 1. While the June/July comparison showed only 80 DEGs, the June/April and July/April comparisons resulted in a considerably higher number of DEGs: 1,682 and 1,822, respectively (Fig. 3A). These results indicated that cork samples of June and July were transcriptionally similar and that differed substantially from those of April. In agreement, in June/April and July/April comparisons 928 were overlapping DEGs and practically all (927) were equally regulated, 529 upregulated and 398 downregulated (Fig. 3A). The expression of all DEGs identified was used to construct a hierarchical clustering of cork samples (Fig. 3B) in which the biological replicates for each month (April, June and July) grouped separately, evidencing a good replicability of the samples. Besides, the clade of June and July samples clustered together, evidencing again a greatest similarity of these months’ transcriptomes compared to those of April.

DEGs transcript profiles of cork samples in April, June and July were grouped in 4 clusters (Fig. 3C). Clusters 2 and 4 contained genes upregulated in April and downregulated in June and July. In contrast, clusters 1 and 3 contained genes downregulated in April and upregulated in June and July, showing cluster 3 a highest variation in gene expression between months and cluster 1 a slight increase in expression in July. Subsequently, GO enrichment analysis was performed to highlight the most important biological processes, molecular functions and cell components within each cluster (Supplemental Tables 5–8).

### Regulatory and abiotic stress genes are induced at the beginning of the growing season (April)

Cluster 2 included the 37.0% of DEGs and was enriched for GO terms associated with (i) response to abiotic stimulus; (ii) metabolic process mainly related with nucleic acid metabolism; (iii) regulation of cell cycle; (iv) transcription factor activity; (v) nucleus compartment; (vi) structural constituent of ribosome and (vii) methyltransferase activity (Supplemental Table 5). The genes in this cluster that were related with GO terms of transcription factor activity, DNA binding and regulation of cell cycle are presented in Supplemental Data Set 2. The set of 114 transcription factors and/or DNA binding proteins were subjected to a secondary GO enrichment analysis to highlight the most important processes in which they were participating. Thirty-four genes were classified in reproductive system development and flower development, 14 in chromosome organization and chromatin organization (6 also in methylation), 9 in meristem development (6 also in meristem maintenance), 5 in cell proliferation, 28 in cell communication, 22 in cell differentiation, 6 in leaf development, 5 in plant epidermis development, 10 in root development and 49 were not included in any of these enriched categories (Supplemental Data Set 2). In Table 1, we highlight some of the candidates to regulate (i) the phellogen meristem identity and maintenance, (ii) the differentiation of phellogen derivatives to phellem cells and (iii) the cell cycle regulation. To narrow those candidates that may have a specific function in phellogen or a shared function with vascular cambium (both secondary tissues), we cross-checked the data with the secondary meristem genes that has been recently described to be upregulated in vascular cambium and/or phellogen/phelloderm of birch tree (*Betula pendula*)^14^, with candidates of phellogen of potato (*Solanum tuberosum*) tuber^18^ and with the candidates for vascular cambium in Arabidopsis root^19^. This information was included in Supplemental Data Set 2. Selected *Q. suber* phellogen candidates discussed in Discussion section are reported in Table 1.

**Table 1:** Selection of candidate genes for phellogen (cork cambium) regulation classified into Meristem development, Cell differentiation and Cell cycle regulation categories that were upregulated at the beginning of the cork growing season (April, in cluster 2). They are sorted by highest expression (Normalized read counts mean) within each category. The complete list is presented in Supplemental Data Set 2.

AGI code	Gene name	Gene function	Differentially expressed *Q. suber* identifier	Normalized read counts mean (BaseMean)	Log_2_ FC June/April (DEG = in bold)	Log_2_ FC July/April (DEG = in bold)
**Meristem development**						
AT4G37750	AINTEGUMENTA (ANT)	Promotion of stem cell proliferation^1^	LOC111985236	5976.93	**−1.67**	−0.73
AT4G28190	ULTRAPETALA1 (ULT1)	Meristem-restricting factor	LOC112024131	5162.97	**−1.34**	**−1.39**
AT4G08150	KNAT1/BREVIPEDICELLUS (BP)	Master regulator of cambial activity^1^ and phellogen regulator	LOC112015571^3^	3784.91	−0.98	−0.84
AT1G46480	WUSCHEL related homeobox 4 (WOX4)	Master regulator of cambial activity^1^ and phellogen regulator	LOC111988759	2676.09	**−1.29**	−0.68
AT1G62360	SHOOT MERISTEMLESS (STM)	Prevents stem cells differentiation^2^	LOC111993990	1942.25	**−1.64**	**−1.56**
AT4G22950	AGAMOUS-like 19 (AGL19)	Regulation of timing of transition from vegetative to reproductive phase	LOC111989313	1622.10	**−1.13**	**−1.45**
AT1G16060	WRINKLED 3 (WRI3)	Positive regulator of cutin and fatty acid biosynthesis^2^	LOC112006985	659.92	−0.51	**−1.28**
AT1G63100	SCARECROW-LIKE 28 (SCL28)	Required for cambial activity^1^	LOC111999471	590.00	**−1.59**	−0.64
AT5G10510	AINTEGUMENTA-like 6 (AIL6)	Promotes of stem cell proliferation	LOC112018242	529.45	**−1.18**	−0.90
AT4G36920	APETALA 2 (AP2)	Promotes the expression of WUS	LOC111995790	528.69	**−1.46**	**−1.38**
AT2G34710	PHABULOSA (PHB)	Stem cell identity maintenance	LOC112031480	485.93	**−1.54**	−1.10
AT2G43000	NAC domain containing protein 42 (NAC042)	Negative regulator of leaf senescence^2^	LOC112006470	211.59	**−1.71**	−0.89
AT2G17950	WUSCHEL (WUS)	Stem cell identity maintenance	LOC111987351	39.35	**−1.85**	−1.12
AT1G31320	LOB domain-containing protein 4 (LBD4)	Cambial regulator of vascular patterning^1^	LOC112038775	13.65	−2.08	**−2.87**
**Cell differentiation**						
AT5G57620	Myb domain protein 36 (MYB36)	Promotion of Casparian Strip lignification and endodermal suberization	LOC112023410	1943.17	−0.63	**−1.06**
			LOC112023896	1875.43	−0.86	**−1.73**
AT3G01140	Myb domain protein 106 (MYB106)	Regulation of cuticular lipid-deposits in epidermal cells	LOC111998628	1329.04	−0.65	**−1.54**
AT4G38620	Myb domain protein 4 (MYB4)	Repressor of phenylpropanoid biosynthesis (lignin and suberin)	LOC111991808	823.27	**−1.10**	−0.43
AT2G23380	CURLY LEAF (CLF)	Polycomb-group protein with repressive activity^4^	LOC111988803	784.64	**−1.36**	**−1.27**
AT1G30970	SUPPRESSOR of FRI 4 (SUF4)	Negative regulation of flower development^4^	LOC111995697	519.78	**−1.10**	−0.69
AT1G68320	Myb domain protein 62 (MYB62)	Unknown	LOC112008278	368.82	**−2.54**	**−1.86**
AT5G41315	GLABROUS 3 (GL3)	Promotion of epidermis differentiation (root hair and nonhair cell fate)	LOC111986568	350.05	**−1.41**	−0.71
AT1G18800	NAP1-related protein 2 (NRP2)	Promotion of cell cycle progression at G2/M and cellular organization, regulation of lateral root formation^4^	LOC111996871	323.02	**−2.13**	**−1.50**
AT3G06740	GATA transcription factor 15 (GATA15)	Unknown	LOC112028517	263.08	**−1.13**	−0.49
AT4G00150	LOST MERISTEMS 3 (LM3)/HAIRY MERISTEM 3 (HAM3)	Promotion of cell differentiation at the periphery of shoot meristems and help to maintain their polar organization.	LOC111988127	199.06	**−1.31**	−0.74
AT4G18710	BRASSINOSTEROID INSENSITIVE 2 (BIN2)	GSK3 targeting the positive regulator of xylem cell differentiation BES1	LOC111992186^5^	121.03	−0.96	0.40
AT5G14750	Myb domain protein 66 (MYB66)/WEREWOLF	Promotion of epidermis differentiation (root hair and nonhair cell fate)	LOC111999826	107.31	**−1.96**	−0.13
AT2G46410	CAPRICE (CPC)	Promotion of epidermis differentiation (root hair and nonhair cell fate)	LOC111988432	106.42	**−2.02**	−0.69
AT4G18390	TEOSINTE BRANCHED 1, cycloidea and PCF transcription factor 2 (TCP2)	Regulation of leaf cell differentiation and secondary shoot formation	LOC112024464	49.96	**−2.32**	−0.63
AT1G22640	Myb domain protein 3 (MYB3)	Repressor of phenylpropanoid biosynthesis (lignin and suberin)	LOC112011850	47.10	**−2.08**	−0.60
AT4G25560	Myb domain protein 18 (MYB18)/LONG AFTER FAR-RED LIGHT 1 (LAF1)	Positive regulation of the PhyA photoresponse	LOC111998979	24.91	**−3.22**	**−3.33**
AT2G31180	Myb domain protein 14 (MYB14)	Unknown	LOC112001589	23.89	−1.34	**−1.82**
AT1G43700	VIRE2-interacting protein 1 (VIP1)/SULPHATE UTILIZATION EFFICIENCY 3 (SUE3)	Negative regulation of cell differentiation, regulation of touch response (thigmotropism)	LOC112021096	14.96	**−2.60**	**−2.94**
**Cell cycle regulation**						
AT4G34160	CYCLIN D3;1 (CYCD3;1)	Involved in the switch from cell proliferation to the final stages of differentiation^1^	LOC112022222	583.60	**−1.87**	−0.33

^1^Involved in vascular cambium development.

^2^Highly expressed in vascular cambium (Zhang et al., 2019).

^3^Not differentially expressed and thus not included in cluster 2, but being at the limit of the threshold (absolute log2Fold Change > 1) to be considered differentially expressed.

^4^Related with chromatin/chromosome organization.

^5^Included in cluster 2 but only DEG in June/July.

Cluster 4 included 11.3% of DEGs and was enriched in GOs associated with (i) response to biotic and abiotic stimulus; (ii) ADP and carbohydrate derivative binding; and (iii) cell periphery (Supplemental Table 6).

### Metabolism and abiotic stress processes dominate in the cork cell transcriptome at the maximum (June) and advanced (July) seasonal growth stages

Cluster 3 included the 40.8% of DEGs and was enriched for GO terms associated with (i) general metabolic processes; (ii) secondary metabolic processes related to lipids, terpenoids, phenylpropanoids and oxylipins; (iii) response to abiotic and biotic stimulus, and in response to salicylic and jasmonic acid; (iv) oxidation–reduction process; (v) chloroplast RNA modification and chloroplast/plastid compartment; (v) ion binding; (vi) kinase activity in relation to ATP and carbohydrate binding; (vii) membrane, especially plasma membrane, but also (viii) cytoplasm (Supplemental Table 7). Interestingly, pathways upstream of suberin, lignin and cork extractives biosynthesis, such as fatty acids, triterpenes, long chain aliphatic compounds, tannins and other aromatic compounds^20^ were all co-upregulated in this cluster.

Cluster 1 included the 10.9% of DEGs and was enriched for GO terms associated with (i) response to stimulus such as light and heat/cold temperature -mostly containing heat shock proteins-, oxidative stress -mainly that generated by hydrogen peroxide-, cadmium, water deprivation and biotic stimulus; (ii) oxidation–reduction process and oxidoreductase activity; (iii) cell wall biogenesis, including lignin and phenylpropanoid metabolism, polysaccharide and carbohydrate-related, the later in agreement with the enriched UDP-glycosyltransferase activity necessary for carbohydrate biosynthesis; (iv) iron and other ion binding; (v) apoplast; (vi) plasma membrane; and (vii) Golgi apparatus, a very active compartment in cell wall matrix polysaccharide biosynthesis and export to the cell wall (Supplemental Table 8). Overall, most of the genes included in this cluster are related to cell wall biogenesis including lignin and polysaccharide biosynthesis and export to the cell wall. Surprisingly, almost 67% (12 from 18) of the genes included in the cell wall biogenesis category were described to contribute to the secondary cell wall thickening of the xylem cells (Table 2) despite that phellem cells, although lignified, does not show the typical secondary cell wall thickenings^4^. The putative involvement of the cell wall candidate genes in the phellem tissue is discussed in the Discussion section.

**Table 2:** *Q. suber* genes in cluster 1 (upregulated in July and/or June) classified within the enriched GOs related with cell wall biogenesis based on the most homologous Arabidopsis gene.

AGI code	Gene name	Gene function	Involved in secondary cell wall biogenesis	Differentially Expressed (july/april) *Q. suber* identifier	Normalized read count mean (BaseMean)	Log2FC July/April
AT4G18780	Cellulose synthase 8 (IRX1)	Cellulose biosynthesis	Yes	LOC112002106	2071.36	2.69
AT5G17420	Cellulose synthase 7 (IRX3)	Cellulose biosynthesis	Yes Yes	LOC112026131	2513.41	2.56
AT5G44030	Cellulose synthase 4 (IRX5)	Cellulose biosynthesis		LOC112023957	871.09	2.45
				LOC111984736	423.38	2.90
AT1G19300	Nucleotide-diphospho-sugar transferases superfamily protein (GALACTURONOSYLTRANSFERASE-LIKE 1, PARVUS)	Glucoronoxylan biosynthesis (hemicellulose). The mutant has thinner secondary cell wall and a reduction of glucuronoxylan	Yes	LOC112011992	659.24	2.21
AT1G27440	Exostosin family protein (IRX10)	Glucuronoxylan biosynthesis (hemicellulose)	Yes	LOC111985053	1245.77	2.05
AT3G18660	Glucuronic acid substitution of xylan 1 (GUX1),Plant glycogenin-like starch initiation protein 1 (PGSIP1)	Glucuronyltransferase adding GlcA residues onto xylan (hemicellulose) and for secondary wall deposition. Also starch biosynthesis	Yes	LOC112008671	278.01	2.86
AT5G54690	Galacturonosyltransferase 12 (IRX8)	The mutant displays reduced xylan (hemicellulose) and cellulose	Yes	LOC112027387	991.26	2.50
AT5G57560	Xyloglucan endotransglucosylase/hydrolase 22 (ATXTH22); Touch 4 (TCH4)	Cell-wall rearrangement; transglycosylation between xyloglucan and xyloglucan-oligosaccharide and analogous reactions (hemicellulose)	–	LOC112028317	1109.07	2.95
AT4G01070	UDP-Glycosyltransferase superfamily protein	Xylosyltransferase	–	LOC112012259^1^	334.20	2.18
				LOC112014189^1^	6907.00	1.88
AT2G38080	Laccase/Diphenol oxidase family protein (IRX12)	Lignin biosynthesis	Yes	LOC112011756	377.32	3.30
				LOC112018093	4900.57	2.65
AT2G29130	Laccase 2	Lignin biosynthesis	–	LOC111992001	1530.80	2.76
AT5G60020	Laccase 17	Lignin biosynthesis, G units	Yes	LOC112004816	91.09	2.67
				LOC112025438	2827.40	3.08
				LOC112025440	322.56	2.30
AT5G09360	Laccase 14	Predicted lignin biosynthesis	–	LOC112039873	7035.98	2.29
AT2G21100	Disease resistance-responsive (dirigent-like protein) family protein	Lignan biosynthetic process	–	LOC112026141^1^	1151.89	3.73
AT2G46770	NAC (No Apical Meristem) domain transcriptional regulator superfamily protein 43 (NST1)	Regulation of secondary wall thickening	Yes	LOC112008346	478.29	2.15
AT5G03170	FASCICLIN-like arabinogalactan-protein 11 (FLA11) (IRX13)	Integrity and elasticity of the plant cell wall matrix	Yes	LOC112031083	1379.34	2.70
AT5G15630	COBRA-like 4, extracellular glycosyl-phosphatidyl inositol (GPI)-anchored protein family (IRX6)	The mutant displays reduced cellulose content, cellulose of lower crystallinity, and thinner secondary cell walls	Yes	LOC112004749	637.38	2.29
AT5G16490	ROP-interactive CRIB motif-containing protein 4	Exocytosis and pollen tube growth	–	LOC111985119	187.41	2.68

^1^Also differentially expressed in June/April comparison.

## Discussion

**Figure 4:**
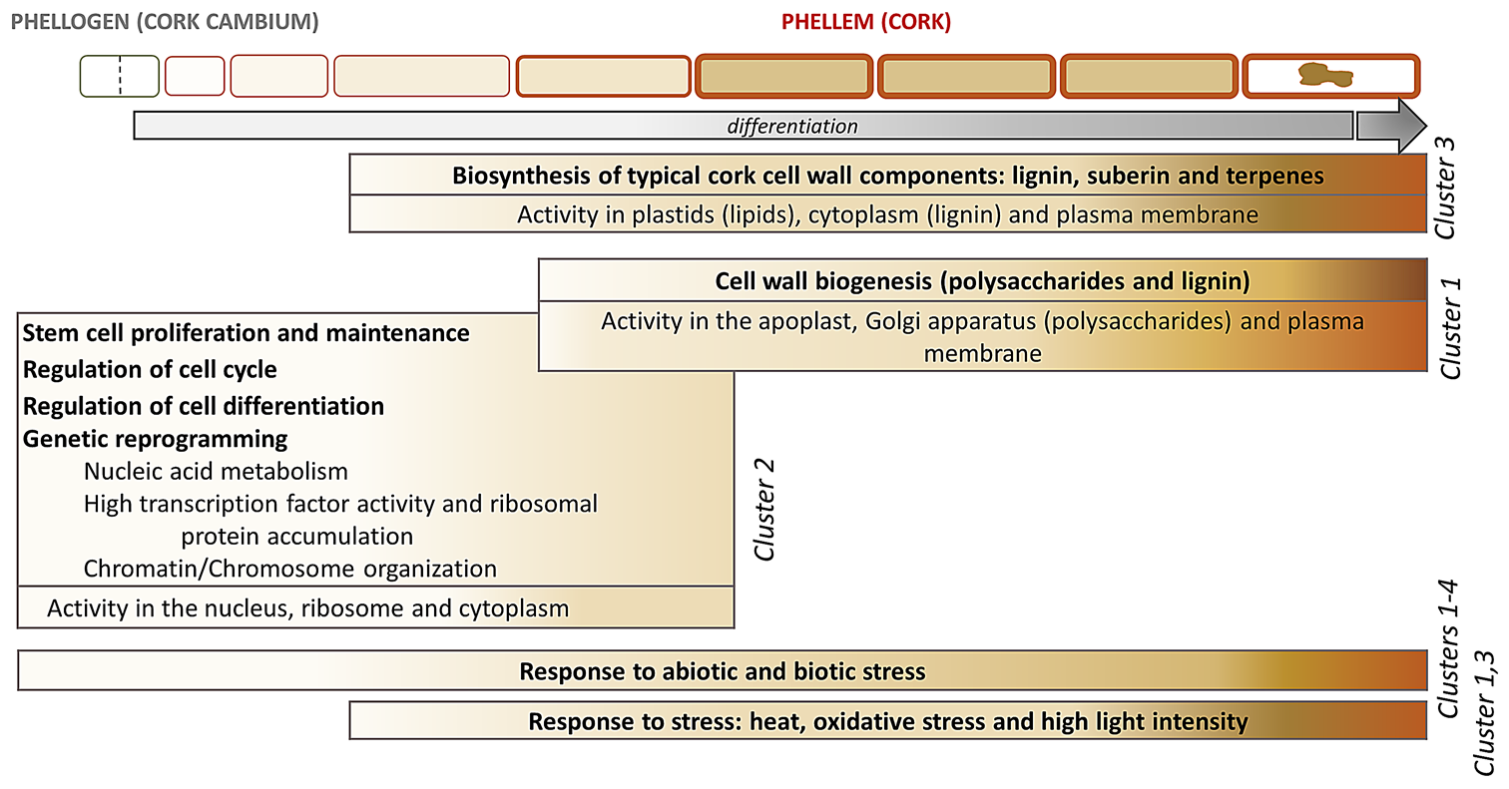
Summary of biological and molecular processes occurring during phellem cell ontogeny, from phellogen stem cell periclinal division to fully differentiated phellem cell, based on upregulated genes and processes in cork samples harvested in April, June and July.

The phellem development is a continuous process in which phellogen cells produces phellogen derivatives outwardly to form fully-developed phellem cells. Due to the seasonal nature of the phellogen growth, the transcriptome profiling along the cork growing season highlighted key molecular processes for the progression of the phellem cell development and overall the formation of cork tissue. The results provide evidences that cell proliferation and cell differentiation is enriched at the beginning of the season while cell wall biogenesis and secondary metabolism are predominant in later stages of cork growth (June and July), and that abiotic stress signalling is a constant factor (Fig. 4).

Regarding phellogen cell proliferation, many genes involved in cell cycle control, such as cyclins, were upregulated in April (cluster 2) (Supplemental Data Set 2), hence indicating that April cork samples were enriched with phellogen cells actively dividing. D-type cyclin CYCD3;1 (Table 1), which is required for vascular cambium-derived secondary thickening, stands out for regulating the cell cycle of phellogen meristematic cells since its promoter is also activated in phellogen compatible cells in Arabidopsis roots^21^, as well as it is highly expressed in potato tuber phellogen^18^.

Cluster 2 was also enriched with genes showing DNA binding capacity, most of them transcription factors (Supplemental Data Set 2 and Table 1). The Meristem development category included genes whose homologs positively regulate the maintenance of the shoot and/or floral meristem and thus they might function similarly in phellogen meristem. From them, WUSCHEL (WUS) maintains stem cell identity^22^ and SHOOT MERISTEMLESS (STM) prevents stem cells from undergoing differentiation^23^. APETALA 2 (AP2) has been described to promote the expression of WUS in floral meristems^24^ and PHABULOSA (PHB), a member of HD-ZIP III family of transcription factors, also maintains stem cells^25^. In floral meristems, AINTEGUMENTA (ANT) and AINTEGUMENTA-LIKE 6 (AIL6) activities have also been suggested to promote continued proliferation of stem cells^26^. Overall, these regulatory genes may converge to functionally maintain phellogen cells in an undifferentiated state. Conversely, Ultrapetala 1 (ULT1), which negatively regulates WUS and acts as a meristem-restricting factor^27^, may contribute to limit the excessive accumulation of cells in phellogen meristem. Interestingly, STM has been suggested to be a potential epigenetic target of the ULT1, that may counteract, as a trithorax-group protein factor, the Polycomb-group repressive activity of the histone methyltransferase CURLY LEAF (CRL), also found in cork transcriptome. The presence of genes involved in Chromosome/Chromatin organization in cluster 2 (Supplemental Data Set 2) reinforce the role of the epigenetic mechanisms in cork development in April which may synchronize the local climate with the transition from dormant to active phellogen growth. Overall, the fact that that meristem-promoting and meristem-restricting factors are enriched in April suggests that overlapping networks may co-exist and adjust phellogen meristem activity and phellogen derivative differentiation in a tight regulatory manner.

Vascular cambium and phellogen both constitute a bifacial stem cell population from which derivatives are specified on opposing sides, in vascular cambium by positional signals^28^. In agreement, some of the genes required for cambium meristematic activity were also upregulated in this cluster 2: AINTEGUMENTA (ANT), WUSCHEL-RELATED HOMEOBOX 4 (WOX4) and SCARECROW-LIKE 28 (SCL28)^19,21,29,30^ (Table 1). Also, the master regulator of vascular cambium activity KNAT1/BREVIPEDICELLUS (BP)^19^ shows high expression with a maximum in April (June/April log_2_FC = -0.98). In agreement with our data and meaningfully, very recently WOX4 and BP has been demonstrated to control phellogen activity in Arabidopsis root standing as periderm-positive regulators^15^, suggesting that phellogen in herbaceous and woody plants may be regulated similarly. Other identified genes have also been observed to be highly expressed in vascular cambium: STM, WRINKLED 3 (WRI3), ANAC042 and LOB DOMAIN-CONTAINING PROTEIN 4 (LBD4), the latter also showing expression in phellogen and involved in vascular patterning^19^.

Many regulators to trigger phellem cell differentiation were also identified upregulated in April (Table 1). We identified LOST MERISTEMS 3 (LM3, also known as HAIRY MERISTEM 3/HAM3) which in shoot apical meristem promotes the differentiation at the periphery^31^, suggesting that in cork it may act similarly by promoting the differentiation of phellem cells outwardly. Also, nine MYB transcription factors were also upregulated in April (Table 1), indicating the importance of this gene family in contributing to phellem cell differentiation. From them, AtMYB106 was described to regulate cuticular lipid-deposits (wax and cutin) in epidermal cells^32^ and three MYBs were related with lignification and/or suberization: AtMYB36 promotes the lignification of the Casparian strip and suberization of endodermis^33,34^, and AtMYB3 and AtMYB4 repress phenylpropanoid biosynthesis^35–37^ and when overexpressed lead to dysfunctional Casparian strip lignification and lower suberin deposition in endodermis and phellem^38^. Overall, these MYB transcription factors are candidates to early regulate in phellogen derivatives to phellem cells the phenylpropanoid and fatty acyl biosynthetic processes that lead to the final accumulation of soluble or insoluble phenylpropanoids (such as condensed tannins or lignin) or fatty-acyl derived compounds (such as waxes or suberin). Also, cluster 2 included a GSK3 protein named BRASSINOSTEROID INSENSITIVE 2 (BIN2; AT4G18710) which regulates xylem cell differentiation^39^. Moreover, three genes upregulated in April were homologs to those which define cell fates in Arabidopsis root epidermis: WEREWOLF/MYB66, GLABROUS 3 and CAPRICE (CPC). Their contribution to phellem formation remains elusive.

During phellem cell development, cells are devoted to synthesize the components that will be deposited in an accurate organization within their cell walls. The major cell wall components of cork (in mass) are: suberin (43%, a glycerol and fatty-acyl-derived polyester including ferulic acid), lignin (22%, a polymer of phenylponane aromatic units), celluloses and hemicellulose (20%, the most important hemicelluloses in cork are xylans) and extractives (16%, including triterpene and phenolic compounds)^40–42^. During the last fifteen years, an intensive work in characterizing biosynthetic genes of suberin, and also extractives, has been carried out in potato tuber phellem but also in other suberizing tissues such as Arabidopsis root endodermis and seed coat, and recently also root phellem (*see* Supplemental Table 4A for citations). From them, the most highly expressed genes showed a peak of expression in June (Fig. 2), an expression profile used to identify new molecular players that participate in the deposition of suberin and associated waxes within the cell wall (Supplemental Figure 2). Recently, three cork oxidosqualene cyclases involved in most cork triterpenoid formation (i.e. friedelin) were biochemically characterized, and the friedelin shynthase also showed a maximum expression in June^43^. This maximum expression in June coincides with the highest cork growth rate^7,17^ and overall aligns with the upregulation of lipid, terpenoid and phenylpropanoid metabolisms in June and July (cluster 3) (Supplemental Table 7).

In contrast to suberin, the molecular processes and functional relevance of lignin and cell wall polysaccharides in phellem, as well as in other suberized cells, is still unclear. Cell wall genes related with lignin and polysaccharide metabolism were enriched in Cluster 1 showing higher expression in June and July but only differentially expressed in July/April comparison (Table 2). Most of these genes were involved in secondary cell wall (SCW) production and included 8 homologous genes (from the 15 identified to date^44^) that when mutated displayed an *irregular xylem* (*irx*) phenotype, characterized for the disruption of the xylem cells, especially vessel cells^45^. The IRX genes correspond to the three cellulose synthase (CESA4/IRX5, CESA7/IRX3 and CESA8/IRX1) required for normal cellulose synthesis in the SCW, three xylan-related hemicellulose biosynthetic genes (*IRX8*, *IRX10* and *IRX15*) specific from SCW production, a lignin (laccase) biosynthetic gene IRX12 and the IRX6/COBRA-LIKE4, a lipid anchored protein with a putative cellulose-binding domain^46–53^. Other genes from Cluster 1 acting on SCW were also involved in xylan biosynthesis (PARVUS and GUX1/PGSIP1) and regulation of wall thickening (NST1/ANAC043)^54,55^. Several lignin-polymerization enzymes specially laccases were also included in this Cluster 1 (Table 2). The identification of genes specific and related with SCW formation was surprising since the phellem or cork cells do not develop a SCW, instead they develop a primary cell wall which inwardly deposits the suberin lamellae and a tertiary cell wall. Possibly, the upregulation of typical SCW genes in July could reproduce the cork-ring anatomy (Fig. 1) since these genes may contribute to the transition from earlycork to latecork cells, which have thicker walls^4^. Hence, we point that typical SCW cellulose, xylan and lignin may participate in the cell wall thickening and biogenesis of latecork formation, providing higher strength, rigidity and hydrophobicity as reported for SCW^56,57^. It is also worth to mention that xylan has been described to be an anchor for lignin deposition, and, in grasses, lignin is often linked to xylan chains via ferulic acids^58,59^, that remarkably is part of the suberin polyester^3^.

Response to abiotic stress is found in all clusters which is in agreement with the ability of ABA to induce suberin accumulation^60,61. ^Genes with higher expression in both June and July (cluster 1 and cluster 3) presented specific enrichment in responses to specific stresses, such as response to light and heat and oxidative stress. The response to light and heat aligns with higher temperature conditions typical of Mediterranean summers, which are reported for the year and area where the cork oaks used for these analyses were growing (Supplemental Figure 3). Oxidative stress in cork samples, and other ligno/suberizing tissues, has been documented^62^ and associated with oxidative coupling of polyphenolic components through a peroxidase/H_2_O_2_ free radical formation^63^. This is in agreement with an enrichment of lignin/suberin accumulation in June and July (Fig. 4).

## Conclusion

The main goal of the current study was to determine the molecular mechanisms and genes that are involved in phellogen to phellem formation. Taking advantage that phellem formation is a continuous developmental process and that cork follows seasonal growth, the comparison of the transcriptome of cork samples harvested at the beginning, maximum and advanced stage of cork formation have highlighted robustly the molecular mechanisms involved in phellogen stem cell proliferation and maintenance, regulation of phellem cell differentiation and phellem cell wall biogenesis. The fact that some of the genes identified have been already characterized in the root periderm of the model plant Arabidopsis reinforces our data, suggests putative shared molecular mechanisms in periderm of both herbaceous and woody species and provide valuable information to assist in the understanding of the general mechanisms controlling phellem protective tissue function.

## Methods

### Plant material and tissue harvesting

The virgin cork (phellem) tissue used was the same used and described by Soler et al*.* (2008)^7^. Cork samples were obtained from trees of 15–20 years old, located in a typical cork oak (*Quercus suber*) forest in Romanyà de la Selva (Girona, Spain) (41°51′42.5″ N, 3°2′7.9″ E; UTM X = 502,951; Y = 4,634,516.2) at a density of 1178 trees/ha. The tree mean diameter was 17.3 ± 5.4 cm and the mean height 8.2 m. Cork samples were collected during the growth period of 2005 (April 26, June 17 and July 19). Climatic data for the 2005 growing season were obtained from the nearby weather station of Castell d’Aro (UTM X = 502,731; Y = 4,628,777), belonging to the Servei Meteorològic de Catalunya (SMC) and are depicted in Supplemental Figure 3. The cork was the first cork stripped from tree trunk (virgin cork), which transcriptome is very similar to that of amadia cork^64^. The tissue harvested was the newly generated and active cork tissue from the belly side of the plank, adjacent to the phellogen tissue. The samples were individually collected from eleven independent trees: four in April, three in June and four in July, immediately frozen in liquid nitrogen and stored at − 80 °C until RNA was extracted.

The virgin cork collection complies with relevant institutional, national, and international guidelines and legislation. For the collection of cork, we obtained the appropriate permission of the Mr. J. Arnedo, the owner of the land where the cork oak forest was located.

## RNA extraction and mRNA purification

Total RNA was extracted from phellem as reported by Soler et al*.* (2008)^7^ using a procedure modified by Chang et al. (1995)^65^. Briefly, 5 g of grinded frozen cork tissue was mixed with extraction buffer (2% CTAB, 2% PVP, 10 mM Tris–HCl pH 8.0, 25 mM EDTA, 2 M NaCl and 2.67% 2-mercaptoethanol) previously heated at 65°C and the mixture was incubated at 65°C for 10 min. Then, two consecutive extractions using 1 volume of chloroform:isoamyl alcohol 24:1 (v/v) were performed, and the RNA in the aqueous phase was precipitated overnight adding 0.25 volumes of 10 M LiCl at 4°C. The precipitate was collected by centrifuging at 12,000 g for 20 min, and the pellet was resuspended in buffer containing 1 M NaCl, 0.5% SDS, 10 mM Tris–HCl pH 8.0 and 1 mM EDTA and incubated at 60 °C for 5 min. Then, one acid phenol:chloroform:isoamyl alcohol (125:24:1 (v/v)) and two 24:1 (v/v) chloroform:isoamyl alcohol extractions were performed. The RNA in the upper phase was precipitated with 2 volumes of 100% ethanol and the precipitate collected by centrifugation. The pellet was washed twice with 70% (v/v) ethanol and resuspended in 50 µl of RNase-free water. RNA extracts were cleaned with the RNAeasy MinElute Cleanup (Qiagen) and genomic DNA was on-column digested with DNase I (Qiagen). The RNA samples sequenced had a RIN (RNA integrity number) value of 8 or higher.

## RNA-seq and bioinformatics analyses

Cork oak cDNA libraries were constructed using the Truseq v4 kit and sequencing was performed by the IlluminaHiSeq2500 platform (paired end reads of 125 bp) at the Ultrasequencing Unit of Center for Genomic Regulation (CRG, Barcelona). In total, 11 samples were sequenced; 3 biological replicates for June and 4 for April and July each. The bioinformatics treatment of raw sequences was performed at the CRG Bionformatic Unit. The quality of reads obtained was evaluated using the FastQC software v0.11.5 (http://www.bioinformatics.babraham.ac.uk/projects/fastqc) and the adapters were eliminated using the Skewer software version 0.2.2^66^. To remove the sequences of polluting ribosomal RNA, the riboPicker version 0.4.3^67^ was used. Then, transcriptome alignment and mapping was made using the STAR_2.5.3a program^68^ against the reference genome GCF_002906115.1 (CorkOak1.0). SAM data was changed to BAM data with samtools 1.4.1^69^. The alignment quality was evaluated using QualiMap v2.2.1 program^70^ and to compare genomic features we used Bedtools v2.26.0^71^. Due to the natural genetic variation of samples, the mapping on cork oak genome was critical to annotate all the corresponding polymorphic alleles of a particular gene of different trees (n = 11) as a unique gene/locus. This allowed to create a matrix of expression at gene/locus level and prevented that specific polymorphic alleles differentially represented among samples were identified as differentially expressed transcripts. For Arabidopsis annotation we used the Blastp and the TAIR10 library from Ensembl, with the options num_alignments 1 and evalue 1E-5.

Raw counts were pre-filtered for low count genes (read count > 1 in every sample) before running the DESeq2 functions^72^ for differential expression analysis between June/April, July/April and June/July comparisons. DEGs were those with an adjusted pvalue ≤ 0.01 and absolute log_2_Fold Change > 1. The normalized read count values for DEGs in at least one comparison were hierarchical clustered using the Pearson correlation through the MeV program and default parameters^73^. Normalized read counts were log transformed (rlog) using DESeq2 to be used for exploratory analysis such as gene expression clustering. The gene clustering was performed using K means clustering in R by imposing 4 clusters, maximum number of iterations = 100,000 and using 10,000 random initial configurations. Expression profiles for each cluster were generated using R (package ggplot2). The grey rows are the expression of every gene, as rlog, while the coloured line is their mean.

Gene ontology (GO) was carried out with AgriGO v.2 (FDR ≤ 0.05)^74^ using the Arabidopsis best homologous gene (TAIR10). The functional GO categories were manually compared, and those with similar GO term description and containing mostly the same set of genes were manually and carefully grouped for a better integration of the data.

The heatmaps of suberin-related genes were constructed with EXPANDERS^75^. To view the expression patterns of the different genes in the same scale the data was standardized as follows: for each gene the mean of the normalized read counts of month’s replicates was log_2_ transformed and the expression pattern (April, June, July) was normalized to have a mean of 0 and a variance of 1. To show an absolute expression value, the sum of normalized read counts for the 11 samples was calculated and expressed as total expression.

For the comparison of the cork transcriptome from cork oak with that of the phellem and phellogen/phelloderm from birch, we used the DEGs in F1/F8 and F2/F8 comparisons from Alonso-Serra et al. (2019)^14^, in which F1 corresponded to phellem, F2 to phellogen and phelloderm and F8 to xylem. For the correspondence, the corresponding Arabidopsis annotations (hits with higher similarity in the BlastP) were used and the duplications were removed from the comparative list.

## Validation of RNA-seq data

RNA-seq results were validated for a subset of 29 representative genes (Supplemental Table 2) by RT-qPCR data generated in previous works using the same samples (22 genes)^7,17^ and 7 new genes, for which the primer sequences are presented in Supplemental Table 9. For RNA-seq and RT-qPCR, the mean of normalized read counts and the mean of relative transcript abundances (RTA) were used, respectively, to calculate the corresponding log_2_Ratio for each comparison (June/April, July/April and July/June). The correlation between RNA-seq and RT-qPCR was assessed by a Pearson correlation analysis.

## Accession numbers

For a comprehensive list of accession numbers of cork oak and their Arabidopsis putative ortholog genes mentioned in the article (mainly discussion), please see Fig. 2, Table 1 and Table 2, as well as Supplemental Data Set 1 and Supplemental Data Set 2.

## Supplementary Information

Supplementary Information 1.

Supplementary Information 2.

Supplementary Information 3.

Supplementary Information 4.

Supplementary Information 5.

Supplementary Information 6.

## Data Availability

The data discussed in this publication have been deposited in NCBI's Gene Expression Omnibus^76^ and are accessible through GEO Series accession number GSE155544 (https://www.ncbi.nlm.nih.gov/geo/query/acc.cgi?acc = GSE155544).

## Abbreviations

DEG: Differentially expressed gene
SCW: Secondary cell wall

## References

[1] Tonn N, Greb T (2017). Radial plant growth. Curr. Biol..

[2] Evert, R. F. Periderm in *Esaus’s Plant Anatomy*. 427–445 (John Wiley & Sons, 2006).

[3] Graça J, Santos S (2007). Suberin: a biopolyester of plants’ skin. Macromol. Biosci..

[4] Pereira H (2007). Cork: Biology, Production and Uses.

[5] Soler M (2007). A genomic approach to suberin biosynthesis and cork differentiation. Plant Physiol..

[6] Caritat, A., Molinas, M. & Gutierrez, E. Annual cork-ring width variability of *Quercus suber* L. in relation to temperature and precipitation (Extremadura, southwestern Spain). *For. Ecol. Manag. ***86**, 113–120 (1996).

[7] Soler, M. *et al.* Seasonal variation in transcript abundance in cork tissue analyzed by real time RT-PCR. *Tree Physiol.* 743–751 (2008). 10.1093/treephys/28.5.743 18316306

[8] Silva SP (2005). Cork: properties, capabilities and applications. Int. Mater. Rev..

[9] Costa A, Pereira H, Oliveira Â (2002). Influence of climate on the seasonality of radial growth of cork oak during a cork production cycle. Ann. For. Sci..

[10] Pereira H, Graça J, Baptista C (1992). The Effect of Growth Rate on the Structure and Compressive Properties of Cork. IAWA J..

[11] Caritat A, Gutiérrez E, Molinas M (2000). Influence of weather on cork-ring width. Tree Physiol..

[12] Rains, M. K., Gardiyehewa de Silva, N. D. & Molina, I. Reconstructing the suberin pathway in poplar by chemical and transcriptomic analysis of bark tissues. *Tree Physiol.* **38**, 340–361 (2018). 10.1093/treephys/tpx060 28575526

[13] Ranathunge K, Schreiber L, Franke R (2011). Suberin research in the genomics era-New interest for an old polymer. Plant Sci..

[14] Alonso-Serra J (2019). Tissue-specific study across the stem reveals the chemistry and transcriptome dynamics of birch bark. New Phytol..

[15] Xiao W (2020). Pluripotent Pericycle Cells Trigger Different Growth Outputs by Integrating Developmental Cues into Distinct Regulatory Modules. Curr. Biol..

[16] Ramos AM (2018). The draft genome sequence of cork oak. Sci. Data.

[17] Boher P (2018). A comparative transcriptomic approach to understanding the formation of cork. Plant Mol. Biol..

[18] Vulavala VKR, Fogelman E, Faigenboim A, Shoseyov O, Ginzberg I (2019). The transcriptome of potato tuber phellogen reveals cellular functions of cork cambium and genes involved in periderm formation and maturation. Sci. Rep..

[19] Zhang J (2019). Transcriptional regulatory framework for vascular cambium development in Arabidopsis roots. Nat. Plants.

[20] Pereira H (1988). Chemical composition and variability of cork from Quercus suber L. Wood Sci. Technol..

[21] Randall RS (2015). AINTEGUMENTA and the D-type cyclin CYCD3;1 regulate root secondary growth and respond to cytokinins. Biol. Open.

[22] Laux T, Mayer KFX, Berger J, Jürgens G (1996). The WUSCHEL gene is required for shoot and floral meristem integrity in Arabidopsis. Development.

[23] Clark SE, Jacobsen SE, Levin JZ, Meyerowitz EM (1996). The CLAVATA and SHOOT MERISTEMLESS loci competitively regulate meristem activity in Arabidopsis. Development.

[24] Huang Z (2017). APETALA2 antagonizes the transcriptional activity of AGAMOUS in regulating floral stem cells in Arabidopsis thaliana. New Phytol..

[25] Pierre-Jerome E, Drapek C, Benfey PN (2018). Regulation of Division and Differentiation of Plant Stem Cells. Annu. Rev. Cell Dev. Biol..

[26] Krizek BA (2009). AINTEGUMENTA and AINTEGUMENTA-LIKE6 act redundantly to regulate arabidopsis floral growth and patterning. Plant Physiol..

[27] Carles CC, Lertpiriyapong K, Reville K, Fletcher JC (2004). The ULTRAPETALA1 gene functions early in Arabidopsis development to restrict shoot apical meristem activity and acts through WUSCHEL to regulate floral meristem determinacy. Genetics.

[28] Smit ME (2020). A PXY-mediated transcriptional network integrates signaling mechanisms to control vascular development in Arabidopsis. Plant Cell.

[29] Ji J (2010). WOX4 promotes procambial development. Plant Physiol..

[30] Suer S, Agusti J, Sanchez P, Schwarz M, Greb T (2011). WOX4 imparts auxin responsiveness to cambium cells in Arabidopsis. Plant Cell.

[31] Schulze S, Schäfer BN, Parizotto EA, Voinnet O, Theres K (2010). LOST MERISTEMS genes regulate cell differentiation of central zone descendants in Arabidopsis shoot meristems. Plant J..

[32] Oshima Y (2013). MIXTA-like transcription factors and WAX INDUCER1/SHINE1 coordinately regulate cuticle development in Arabidopsis and Torenia fournieri. Plant Cell.

[33] Kamiya T (2015). The MYB36 transcription factor orchestrates Casparian strip formation. Proc. Natl. Acad. Sci. USA.

[34] Wang C (2020). Developmental programs interact with abscisic acid to coordinate root suberization in Arabidopsis. Plant J..

[35] Jin H (2000). Transcriptional repression by AtMYB4 controls production of UV-protecting sunscreens in Arabidopsis. EMBO J..

[36] Dubos C (2010). MYB transcription factors in Arabidopsis. Trends Plant Sci..

[37] Fornalé S (2014). AtMYB7, a New Player in the Regulation of UV-Sunscreens in Arabidopsis thaliana. Plant Cell Physiol..

[38] Andersen TG (2021). Tissue-autonomous phenylpropanoid production is essential for establishment of root barriers. Curr. Biol..

[39] Kondo Y (2014). Plant GSK3 proteins regulate xylem cell differentiation downstream of TDIF-TDR signalling. Nat. Commun..

[40] Conde E, Cadahía E, Garcia-Vallejo MC, Gonźalez-Adrados JR (1998). Chemical Characterization of Reproduction Cork from Spanish Quercus Suber. J. Wood Chem. Technol..

[41] Conde, E., Cadahía, E., García-Vallejo, M. C., Fernández de Simón, B. & González Adrados, J. R. Low molecular weight polyphenols in cork of *Quercus suber*. *J. Agric. Food Chem.* **45**, 2695–2700 (1997).

[42] Pereira H (2013). Variability of the chemical composition of cork. BioResources.

[43] Busta L (2020). Oxidosqualene cyclases involved in the biosynthesis of triterpenoids in Quercus suber cork. Sci. Rep..

[44] Hao Z, Mohnen D (2014). A review of xylan and lignin biosynthesis: Foundation for studying Arabidopsis irregular xylem mutants with pleiotropic phenotypes. Crit. Rev. Biochem. Mol. Biol..

[45] Turner SR, Somerville CR (1997). Collapsed xylem phenotype of arabidopsis identifies mutants deficient in cellulose deposition in the secondary cell wall. Plant Cell.

[46] Liu L (2013). Brittle Culm1, a COBRA-Like Protein, Functions in Cellulose Assembly through Binding Cellulose Microfibrils. PLoS Genet..

[47] Persson S, Wei H, Milne J, Page GP, Somerville CR (2005). Identification of genes required for cellulose synthesis by regression analysis of public microarray data sets. Proc. Natl. Acad. Sci. USA.

[48] Brown DM, Zeef LAH, Ellis J, Goodacre R, Turner SR (2005). Identification of novel genes in Arabidopsis involved in secondary cell wall formation using expression profiling and reverse genetics. Plant Cell.

[49] Brown DM, Zhang Z, Stephens E, Dupree P, Turner SR (2009). Characterization of IRX10 and IRX10-like reveals an essential role in glucuronoxylan biosynthesis in Arabidopsis. Plant J..

[50] Brown D (2011). Arabidopsis genes IRREGULAR XYLEM (IRX15) and IRX15L encode DUF579-containing proteins that are essential for normal xylan deposition in the secondary cell wall. Plant J..

[51] Peña MJ (2007). Arabidopsis irregular xylem8 and irregular xylem9: Implications for the complexity of glucuronoxylan biosynthesis. Plant Cell.

[52] Jensen JK (2011). The DUF579 domain containing proteins IRX15 and IRX15-L affect xylan synthesis in Arabidopsis. Plant J..

[53] Wu AM (2009). The Arabidopsis IRX10 and IRX10-LIKE glycosyltransferases are critical for glucuronoxylan biosynthesis during secondary cell wall formation. Plant J..

[54] Lee C (2007). The PARVUS Gene is Expressed in Cells Undergoing Secondary Wall Thickening and is Essential for Glucuronoxylan Biosynthesis. Plant Cell Physiol..

[55] Mortimer JC (2010). Absence of branches from xylan in Arabidopsis gux mutants reveals potential for simplification of lignocellulosic biomass. Proc. Natl. Acad. Sci. USA.

[56] Scheller HV, Ulvskov P (2010). Hemicelluloses. Annu. Rev. Plant Biol..

[57] Vanholme R, Demedts B, Morreel K, Ralph J, Boerjan W (2010). Lignin biosynthesis and structure. Plant Physiol..

[58] Grabber JH, Ralph J, Hatfield RD (2002). Model studies of ferulate—Coniferyl alcohol cross-product formation in primary maize walls: Implications for lignification in grasses. J. Agric. Food Chem..

[59] Gregory ACE, Smith C, Kerry ME, Wheatley ER, Bolwell GP (2002). Comparative subcellular immunolocation of polypeptides associated with xylan and callose synthases in French bean (Phaseolus vulgaris) during secondary wall formation. Phytochemistry.

[60] Barberon M (2016). Adaptation of root function by nutrient-induced plasticity of endodermal differentiation. Cell.

[61] Lulai EC, Suttle JC, Pederson SM (2008). Regulatory involvement of abscisic acid in potato tuber wound-healing. J. Exp. Bot..

[62] Pla M (1998). Stress proteins co-expressed in suberized and lignified cells and in apical meristems. Plant Sci..

[63] Razem FA, Bernards MA (2003). Reactive oxygen species production in association with suberization: evidence for an NADPH-dependent oxidase. J. Exp. Bot..

[64] Lopes ST (2020). Phellem versus xylem: Genome-wide transcriptomic analysis reveals novel regulators of cork formation in cork oak. Tree Physiol..

[65] Chang S, Puryear J, Cairney J (1993). A simple and efficient method for isolating RNA from pine trees. Plant Mol. Biol. Report..

[66] Jiang H, Lei R, Ding S-W, Zhu S (2014). Skewer: a fast and accurate adapter trimmer for next-generation sequencing paired-end reads. BMC Bioinformatics.

[67] Schmieder R, Lim YW, Edwards R (2012). Identification and removal of ribosomal RNA sequences from metatranscriptomes. Bioinformatics.

[68] Dobin A (2013). STAR: ultrafast universal RNA-seq aligner. Bioinformatics.

[69] Li H (2009). The Sequence Alignment/Map format and SAMtools. Bioinformatics.

[70] García-Alcalde F (2012). Qualimap: evaluating next-generation sequencing alignment data. Bioinformatics.

[71] Quinlan AR, Hall IM (2010). BEDTools: a flexible suite of utilities for comparing genomic features. Bioinformatics.

[72] Love MI, Huber W, Anders S (2014). Moderated estimation of fold change and dispersion for RNA-seq data with DESeq2. Genome Biol..

[73] Howe, E. *et al.* MeV: MultiExperiment Viewer in *Biomedical Informatics for Cancer Research* 267–277 (Springer, 2010).

[74] Tian, T. *et al.* agriGO v2.0: a GO analysis toolkit for the agricultural community, 2017 update. *Nucleic Acids Res.* **45**, W122–W129 (2017). 10.1093/nar/gkx382 PMC5793732 28472432

[75] Ulitsky I (2010). Expander: from expression microarrays to networks and functions. Nat. Protoc..

[76] Edgar R, Domrachev M, Lash AE (2002). Gene Expression Omnibus: NCBI gene expression and hybridization array data repository. Nucleic Acids Res..

