# Supplementary material for: Transcriptomic analysis of cork during seasonal growth highlights regulatory and developmental processes from phellogen to phellem formation
Source: Sci Rep. 2021 Jun 8;11:12053. doi: 10.1038/s41598-021-90938-5 (PMC8187341; doi:10.1038/s41598-021-90938-5)
Supplement: Supplementary file 1 — Supplementary Information 1. [file 41598_2021_90938_MOESM1_ESM.pdf]

Supplemental figure file for:

Transcriptomic analysis of cork during seasonal growth highlights regulatory and developmental processes from phellogen to phellem formation

Sandra Fernández-Piñán[1], Pau Boher[1], Marçal Soler[1], Mercè Figueras[1] and Olga Serra[1*]

[1]Laboratori del Suro, Departament de Biologia, Universitat de Girona, Campus Montilivi, E-17003 Girona, Spain.

[*]corresponding author: Olga Serra, olga.serra@udg.edu

Supplemental Tables 3-8 and Supplemental datasets 1 and 2 are presented in separated Excel files.

Supplemental Figure 1

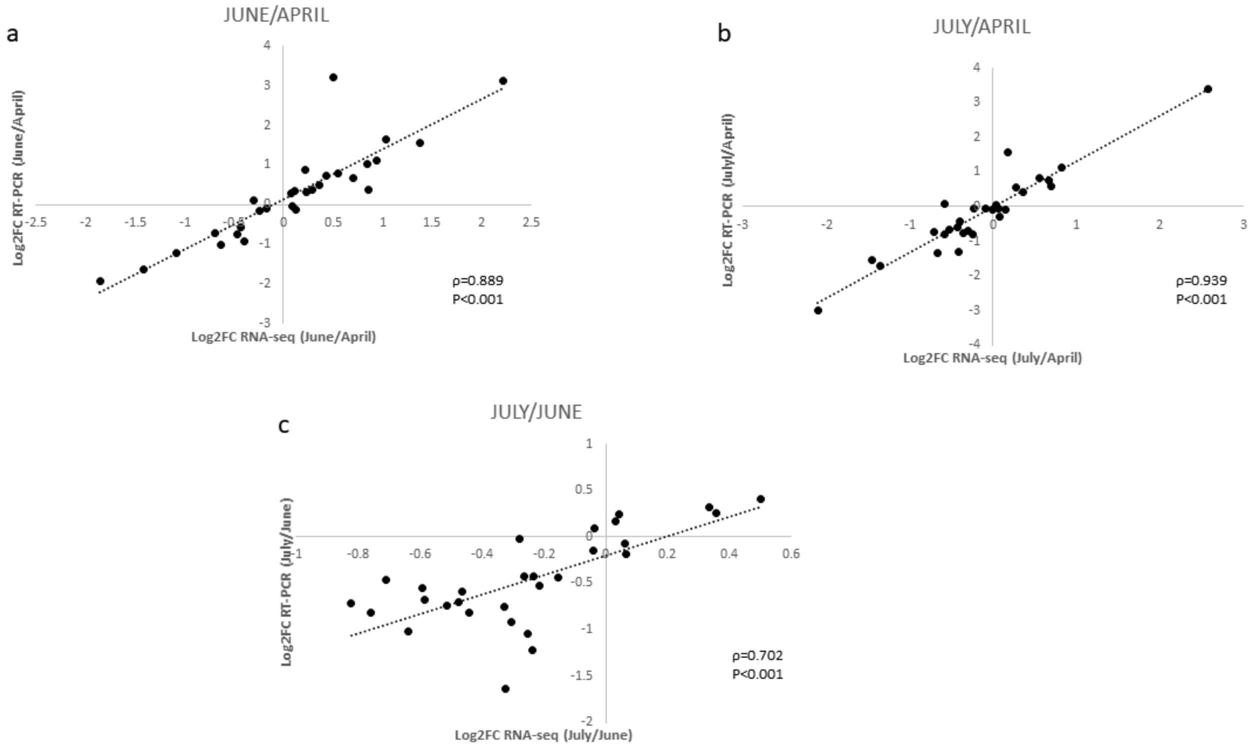

Supplemental Figure 1. Correlation between $\log_2$Fold-Change data obtained in the RT-qPCR and RNA-seq experiments. The linear regression with the Pearson correlation (ρ) is represented. Correlation results obtained for comparison between the data obtained in June/April (a), July/April (b) and July/June (c). The data and genes under study are presented in Supplemental Table 2.

Supplemental Figure 2

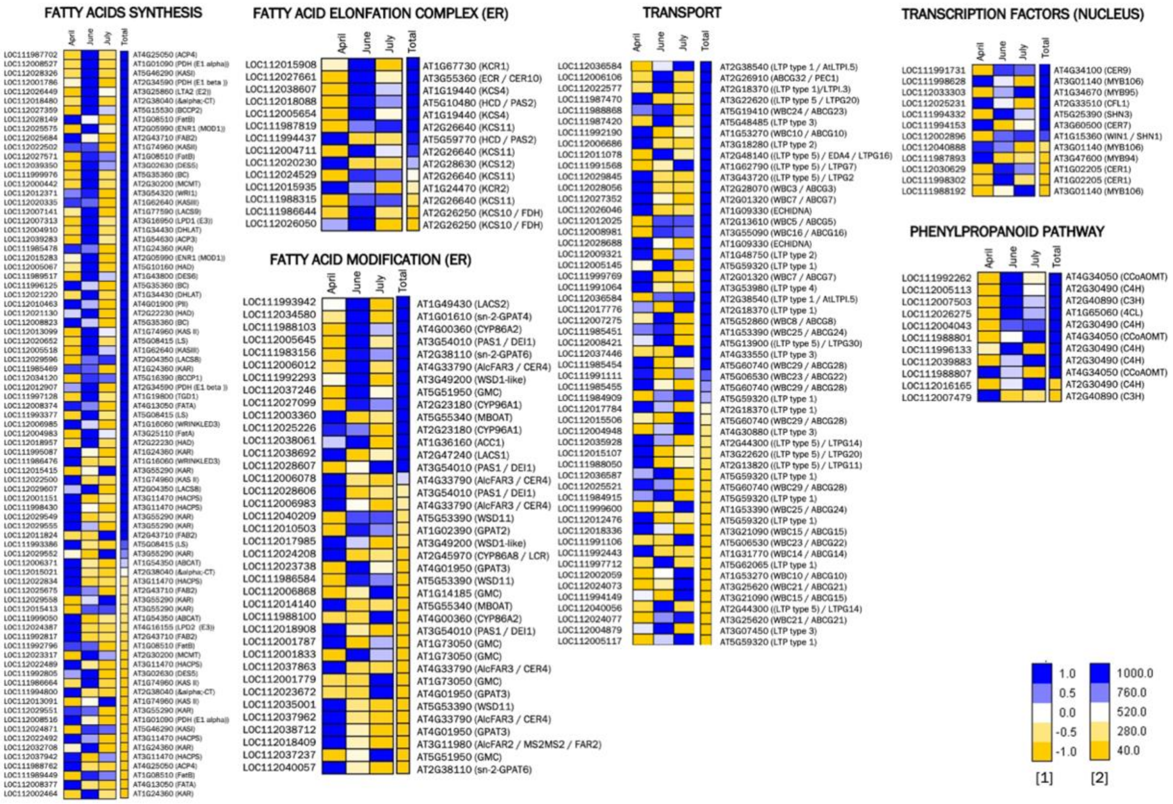

Supplemental Figure 2. Expression pattern along the cork growing season of AraLip genes not related previously with suberization process. For each gene, the relative expression pattern (April, June and July) [1] and the total expression [2] of normalized read counts are shown as a heatmap, in which blue and yellow colours represent high and low expression values. ER = endoplasmic reticulum. The heatmaps were constructed using the EXPANDERS software[75].

Supplemental Figure 3

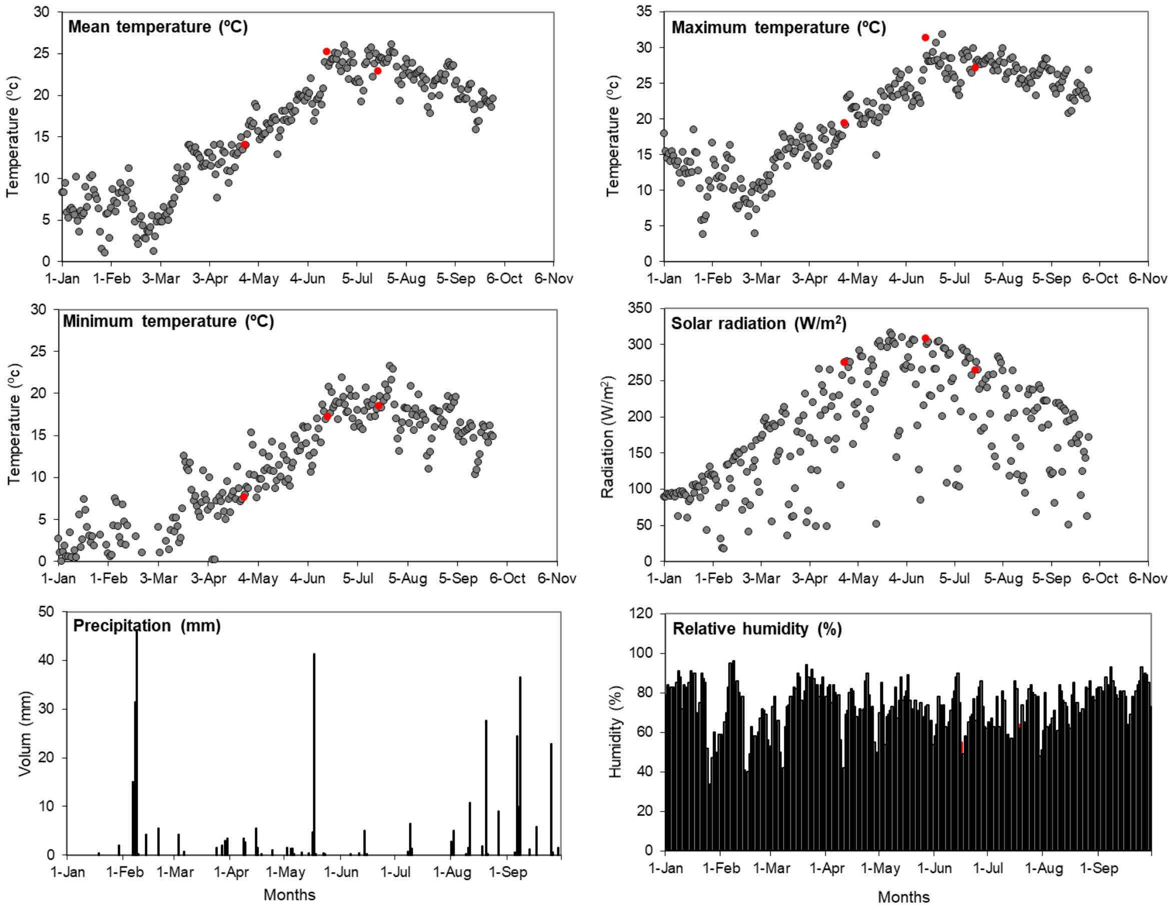

Supplemental Figure 3. Climatic conditions in the region where the samples where collected, from January to September 2005. The mean, maximum and minimum temperature (ºC), solar radiation (W m^{-2}), precipitation (mm), and relative humidity (%) are depicted. The data points corresponding to the days when the samples under analyses were collected are highlighted in red.

Supplemental Table 1. Metrics of RNA-seq data.

Sample name	FastQC trimmed sequences				Mapping against GCF_002906115.1 (CorkOak1.0)			Functional annotation Arabidopsis
	Average GC content (%)	Total reads (millions - 10^6)	Average trimmed sequences (%)	Average read length (bp)	Uniquely mapped reads (millions - 10^6)	Average uniquely mapped reads (%)	Total Q. suber loci mapped	BLASTX hit TAIR10 (Arabidopsis protein Db)
**Total**	**45.1**	**568.3**	**11.9**	**122.0**	**464.7**	**81.3**	**32024**	**30676**
April_1	44	58.7	9.5	122.0	50.5	86.1		
April_2	45	51.8	11.6	122.0	43.3	83.7		
April_3	44	57.7	11.9	122.0	48.5	84.1		
April_4	44	68.3	16.4	122.0	56.9	83.4		
June_1	46	40.4	12.9	122.0	31.4	77.7		
June_3	45	73.4	11.2	122.0	60.9	83.0		
June_4	45	52.1	9.7	122.0	42.9	82.4		
July_1	47	40.2	15.5	122.0	27.4	68.3		
July_2	46	48.5	10.8	122.0	39.0	80.4		
July_3	45	36.9	11.4	122.0	30.8	83.4		
July_4	45	40.3	9.5	122.0	33.1	82.1		

Supplemental Table 2. Gene expression values (log$_2$Fold-Change) of RNA-seq and qRT-PCR (RTA) data used to validate the RNA-seq analysis.

					JUNE/APRIL		JULY/APRIL		JULY/JUNE	
**Category**	**ID *Quercus***	**ID Arabidopsis**	**Gene Name**	**DESCRIPTIONS**	**Log2RNA-seq**	**Log2RTA[1]**	**Log2RNA-seq**	**Log2RTA[1]**	**Log2RNA-seq**	**Log2RTA[1]**
Carbohydrate metabolism	LOC112019923	AT3G43190	SUS4	*Sucrose Synthase 4*	0.290	0.373	0.059	-0.053	-0.232	-0.426
Fatty acids synthesis	LOC112030007	AT1G01090	PDH-E1α[2]	*Pyruvate Dehydrogenase e1 alpha*	0.432	0.722	-0.010	-0.093	-0.442	-0.815
	LOC112012371	AT3G54320	WRI1[2]	*WRINKLED 1*	0.114	0.35	-0.526	-0.664	-0.639	-1.014
	LOC112028149	AT1G08510	FATB	*Fatty Acyl-ACP Thioesterases B*	-0.695	-0.73	-1.454	-1.545	-0.759	-0.814
Cell wall	LOC112019461	AT2G40610	EXPA8	*Expansin A8*	0.842	1.033	0.690	0.589	-0.153	-0.444
Hormone metabolism	LOC111999928	AT3G13380	BRL3[2]	*BRI1-like 3*	0.237	0.31	0.281	0.556	0.045	0.246
	LOC112022266	AT1G70940	PIN3	*PIN-formed 3*	-0.637	-1.022	-0.304	-0.699	0.333	0.323
	LOC112001486	AT3G63010	GID1B[2]	*GA Insentitive DWARF 1B*	0.499	3.206	0.174	1.566	-0.325	-1.64
Cell death	LOC111985828	AT5G47120	BI1	*BAX inhibidor 1*	0.856	0.366	0.147	-0.092	-0.709	-0.458
Meristem regulation	LOC111993884	AT3G23150	ETR2[2]	*Ethylene response 2*	0.218	0.874	-0.087	-0.038	-0.306	-0.912
	LOC111994940	AT3G19820	DWF1	*DWARF 1*	-0.429	-0.564	-0.363	-0.753	0.066	-0.19
	LOC112030007	AT1G27320	HK3[2]	*Histidine Kinase 3*	-0.306	0.117	-0.584	0.097	-0.278	-0.02
	LOC111993990	AT1G36062	STM	*Shoot Meristemless*	-1.413	-1.65	-1.351	-1.722	0.062	-0.072
	LOC111988759	AT1G46480	WOX4	*Wuschel Related Homeobox 4*	-1.078	-1.212	-0.576	-0.806	0.501	0.406
	LOC112029705	AT4G32880	HB8[2]	*Homeobox Gene 8*	1.032	1.64	0.817	1.117	-0.216	-0.523
Transcription factors	LOC111990209	AT2G46130	WRKY43	*WRKY DNA-Binding protein 43*	0.084	-0.031	-0.244	-0.783	-0.328	-0.753
	LOC112037487	AT3G18400	NAC058	*NAC Domain containing protein 58*	0.079	0.284	-0.398	-0.415	-0.477	-0.699
MADS-box proteins	LOC112015273	AT5G10140	FLC	*Flowering Locus C*	-0.400	-0.924	-0.665	-1.349	-0.265	-0.426
	LOC112012679	AT2G45660	SOC1	*Supressor of Overexpression of CO 1*	-0.247	-0.15	-0.709	-0.745	-0.462	-0.594
	LOC111991050	AT1G69120	AP1	*Apetala 1*	-0.176	-0.093	-0.411	-1.314	-0.235	-1.221
Suberin synthesis	LOC112007140	AT5G41040	ASFT/HCBT	*N-Hydroxycinnamoyl/ Benzoyl Transferase*	0.550	0.775	0.036	0.039	-0.514	-0.735
	LOC112038977	AT5G58860	CYP86A1	*Cytochrome P450 Family 86 Subfamily A Polypeptide 1*	0.936	1.108	0.352	0.436	-0.584	-0.672
	LOC112028279	AT3G11430	GPAT5	*Glycerol-3-Phosphatase sn-2-acyl Transferase 5*	0.357	0.5	-0.235	-0.046	-0.591	-0.546
Secondary metabolism	LOC111989132	AT1G61720	BAN	*Barely Any Meristem*	-0.466	-0.762	-0.434	-0.591	0.032	0.171
	LOC112000640	AT1G78955	bAS	*β-Amyrin Synthase*	1.381	1.55	0.557	0.839	-0.824	-0.711
	LOC111994345	AT4G36220	F5H	*Ferulate-5-hydrolase*	-1.853	-1.945	-2.104	-2.988	-0.251	-1.043
Stress	LOC112002958	AT5G59720	HSP17.4	*Heat Shock Protein 17.4*	2.217	3.124	2.573	3.381	0.356	0.256
	LOC112011353	AT1G35720	ANN	*Annexin*	0.701	0.681	0.667	0.768	-0.035	0.087
	LOC112028339	AT3G09640	APX2	*Ascorbate peroxidase*	0.121	-0.141	0.083	-0.285	-0.038	-0.143

[1] Calculated using the data from Soler et al. (2008) and Boher et al. (2018)

[2] Genes analysed in this work, the primers sequences are detailed in Supplemental Table 9.

Supplemental Table 9. Primer sequences of the 9 genes that were analysed by RT-qPCR analyses in this work.

Gene Name	*Quercus suber* identifiers	Best Arabidopsis homologous gene identifier	Primer sequences (from 5'- to 3'-end), forward (Fwd) and reverse (Rev) primers
PDH-E1α	LOC112030007	AT1G01090	Fwd: GGTTAGGGAGGTGGCAAAGG<br>Rev: TTCTCAGCAGGGTCACGAAG
WRI1	LOC112012371	AT3G54320	Fwd: GGCATTTCTCTGGGTTGTGA<br>Rev: CCTGTGTTGGTTCTGTCATTCC
BRL3	LOC111999928	AT3G13380	Fwd: AGAAAGCAAAGGCGAAGGAA<br>Rev: TGGGGAGAACAAAGTAATGGAG
GID1B	LOC112001486	AT3G63010	Fwd: AAGCCCTCCAATACCAATCG<br>Rev: TCCTTCTTCATCCAATGTTTGC
ETR2	LOC111993884	AT3G23150	Fwd: AAGGGGAGCGATGAAGTGAA<br>Rev: ATTGGAAACCCGAAGCATTG
HK3	LOC112030007	AT1G27320	Fwd: CGACATCAAAGACACCACCA<br>Rev: TCTCCCCTCAAATGCTACCC
HB8	LOC112029705	AT4G32880	Fwd: CAGCAGTTCCAGTAGCCTTCG<br>Rev: TCAACACCATCTGACACCTCA
